# Azepine-Indole Alkaloids From *Psychotria nemorosa* Modulate 5-HT_2*A*_ Receptors and Prevent *in vivo* Protein Toxicity in Transgenic *Caenorhabditis elegans*

**DOI:** 10.3389/fnins.2022.826289

**Published:** 2022-03-14

**Authors:** Benjamin Kirchweger, Luiz C. Klein-Junior, Dagmar Pretsch, Ya Chen, Sylvian Cretton, André L. Gasper, Yvan Vander Heyden, Philippe Christen, Johannes Kirchmair, Amélia T. Henriques, Judith M. Rollinger

**Affiliations:** ^1^Department of Pharmaceutical Sciences, Division of Pharmacognosy, University of Vienna, Vienna, Austria; ^2^School of Health Sciences, Universidade do Vale do Itajaí (UNIVALI), Itajaí, Brazil; ^3^Laboratory of Pharmacognosy and Quality Control of Phytomedicines, Faculty of Pharmacy, Universidade Federal do Rio Grande do Sul (UFRGS), Porto Alegre, Brazil; ^4^Department of Pharmaceutical Sciences, Division of Pharmaceutical Chemistry, University of Vienna, Vienna, Austria; ^5^Institute of Pharmaceutical Sciences of Western Switzerland, University of Geneva, Geneva, Switzerland; ^6^Herbarium Dr. Roberto Miguel Klein, Department of Natural Sciences, Universidade Regional de Blumenau (FURB), Blumenau, Brazil; ^7^Department of Analytical Chemistry, Applied Chemometrics and Molecular Modeling, Center for Pharmaceutical Research (CePhaR), Vrije Universiteit Brussel (VUB), Brussels, Belgium

**Keywords:** bioactive natural products, molecular target prediction, alkaloids, *Psychotria nemorosa*, *Caenorhabditis elegans*, 5-HT_2A_, neurodegeneration

## Abstract

Nemorosine A (**1**) and fargesine (**2**), the main azepine-indole alkaloids of *Psychotria nemorosa*, were explored for their pharmacological profile on neurodegenerative disorders (NDs) applying a combined *in silico*–*in vitro*–*in vivo* approach. By using **1** and **2** as queries for similarity-based searches of the ChEMBL database, structurally related compounds were identified to modulate the 5-HT_2*A*_ receptor; *in vitro* experiments confirmed an agonistic effect for **1** and **2** (24 and 36% at 10 μM, respectively), which might be linked to cognition-enhancing properties. This and the previously reported target profile of **1** and **2**, which also includes BuChE and MAO-A inhibition, prompted the evaluation of these compounds in several *Caenorhabditis elegans* models linked to 5-HT modulation and proteotoxicity. On *C. elegans* transgenic strain CL4659, which expresses amyloid beta (Aβ) in muscle cells leading to a phenotypic paralysis, **1** and **2** reduced Aβ proteotoxicity by reducing the percentage of paralyzed worms to 51%. Treatment of the NL5901 strain, in which α-synuclein is yellow fluorescent protein (YFP)-tagged, with **1** and **2** (10 μM) significantly reduced the α-synuclein expression. Both alkaloids were further able to significantly extend the time of metallothionein induction, which is associated with reduced neurodegeneration of aged brain tissue. These results add to the multitarget profiles of **1** and **2** and corroborate their potential in the treatment of NDs.

## Introduction

Indian tribes from Amazonia have been using leaves from *Psychotria viridis* (in combination with *Banisteriopsis caapi*) for the preparation of Ayahuasca, a decoction applied in rituals for the treatment of various health problems, including mental diseases (Katchborian-Neto et al., 2020). Since the early 1990s, research groups have investigated the use of Ayahuasca for the treatment of anxiety, depression, alcohol addiction, and neurodegenerative diseases (NDs) (Domínguez-Clavé et al., 2016; Dos Santos and Hallak, 2021). The pharmacological properties of Ayahuasca are attributed to the indole alkaloids identified in the beverage: tryptamine derivatives from *P. viridis* and β-carboline derivatives from *B. caapi* (Katchborian-Neto et al., 2020). Stimulated by the research on Ayahuasca, further *Psychotria* species became the subject of pharmacological and pharmacobotanical studies. Several indole alkaloids have been isolated and evaluated regarding their bioactivity on proteins related to Alzheimer’s disease (AD) and Parkinson’s disease (PD) (Passos et al., 2013; dos Santos Passos et al., 2015; Klein-Júnior et al., 2016, 2017, 2020).

Alzheimer’s disease and PD are multifactorial NDs for which the etiology is poorly understood (AlDakheel et al., 2014; Sanabria-Castro et al., 2017; Nimmo et al., 2021). AD is characterized by cognitive impairment whereby neurotoxic Aβ oligomers contribute to its onset and progression (Lambert et al., 1998). Commonly applied medicines include cholinesterase inhibitors, e.g., donepezil, galantamine, and rivastigmine, the *N*-methyl-D-aspartate-receptor-antagonist memantine, and *Ginkgo biloba* extract EGb 761 (Liu et al., 2015; Sharma et al., 2019; Nowak et al., 2021). However, none of these therapeutics is able to alleviate the causative disorder, but only delay the progression of symptoms related to AD. For PD, the situation is similar. PD is characterized by motor deficits caused by the degeneration of dopaminergic neurons mediated by α-synuclein aggregates. Therefore, several dopaminergic therapies are used as symptomatic treatment, e.g., levodopa (dopamine precursor), selegiline (monoamine oxidase B inhibitor), entacapone (catechol *O*-methyltransferase inhibitor), and pramipexole (dopamine agonist) (Poewe et al., 2017; Ntetsika et al., 2021).

In recent years, the drug discovery paradigm has shifted from single-target pharmacology to the recognition and utilization of multitarget pharmacology, whereby clinical activity is expressed as the sum of several pharmacodynamic effects on multiple targets (Chaudhari et al., 2017). With increasing knowledge of drug–target interactions, more effective drugs, which modulate multiple targets, might be discovered for diseases such as PD and AD (Wang et al., 2019; Mok et al., 2020; Paul et al., 2020; Zagórska and Jaromin, 2020; Albertini et al., 2021).

**FIGURE 1:**
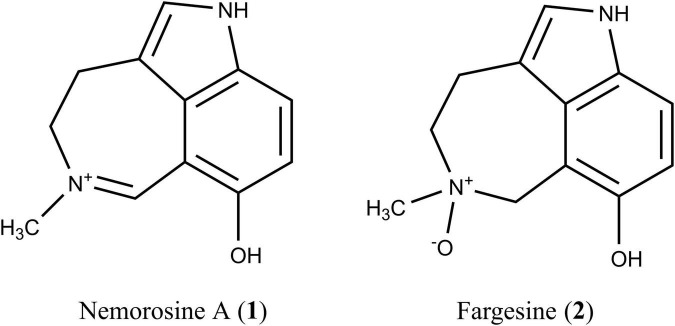
Structures of the *Psychotria nemorosa* alkaloids nemorosine A (**1**) and fargesine (**2**).

Recently, it was demonstrated that the alkaloid fraction obtained from the leaves of *Psychotria nemorosa* Gardner (PNAF) is able to inhibit the activities of butyrylcholinesterase (BuChE) and monoamine oxidase A (MAO-A) (Klein-Júnior et al., 2016, 2020). Chemical analysis led to the isolation of 10 azepine-indole alkaloids, most of them with a cinnamoyl and/or a secologanin moiety. However, due to the bulkiness of these glucosylated alkaloids, they were not able to inhibit either BuChE or MAO-A. On the other hand, smaller alkaloids obtained from PNAF, including nemorosine A (**1**) and fargesine (**2**) (Figure 1), inhibited both targets (Klein-Júnior et al., 2020).

This study has two objectives:

(i) To broaden the knowledge on the potential multitarget effect of representative alkaloids from *P. nemorosa*: thus, an *in silico* study was performed to predict the bioactivity profiles of the main alkaloids **1** and **2**.
(ii) To gain insight into the effects of the multitarget profile of **1** and **2** *in vivo*; thus, the compounds were tested in the model organism *Caenorhabditis elegans* assessing different phenotypic readouts, namely, locomotion, pharyngeal pumping, fat accumulation, AD/PD proteotoxicity, and metallothionein (MT) expression.

*Caenorhabditis elegans* is a nematode with a rapid life cycle and transparent body (ideal for fluorescent markers), whose genome is fully sequenced with 60–80% homology with human genes. These features make *C. elegans* a suitable model to study human diseases (Hulme and Whitesides, 2011; Markaki and Tavernarakis, 2020). The serotoninergic signaling system is largely conserved between nematodes and humans (Alcedo and Prahlad, 2020; Ishita et al., 2020). 5-HT regulates a wide range of *C. elegans* behavior like pharyngeal pumping (Carre-Pierrat et al., 2006), locomotion (Gürel et al., 2012), and egg laying (Dempsey et al., 2005). It influences energy balance (Srinivasan et al., 2008), lifespan, learning, and behavioral aging (Murakami and Murakami, 2007; Murakami et al., 2008). *C. elegans* has three functioning cholinesterase classes, whereby ACE-3 also has BuChE activity. The ACE-3 receptor is responsible for approximately 5% of overall acetylcholinesterase activity of the worm and is mainly expressed in the pharynx (Combes et al., 2000; Melstrom and Williams, 2007).

The applicability of transgenic *C. elegans* to study proteotoxicity-related diseases, such as AD and PD, has been highlighted previously (Liu et al., 2015; Paul et al., 2020). In this study, three transgenic *C. elegans* strains were used as models: (1) Strain CL2659 expressing Aβ in muscle cells shows a paralysis phenotype after 48 h at 25°C and was applied to study treatments that encounter soluble Aβ oligomerization leading to a delayed onset of the paralyzed phenotype. (2) Strain NL5901 was used to monitor the expression of its YFP-tagged α-synuclein by a fluorescent reader. (3) Strain CL2120 with green fluorescent protein (GFP)-tagged MT and constitutively expressed Aβ was applied to investigate a functional mode of action. MT is an intracellular, low-molecular, cysteine-rich protein participating in the transport, homeostasis, and detoxification of heavy metals. Its expression and regulation is related to progressive NDs (Pretsch et al., 2020).

Here we demonstrate that **1** and **2** are not only inhibitors of MAO-A and BuChE. *In vitro* results obtained in human embryonic kidney (HEK-293) cells mainly point toward a 5-HT_2*A*_ receptor agonistic activity. In the nematode assays, however, both alkaloids showed the behavioral profile of 5-HT antagonists. Moreover, they showed a distinct positive effect on the AD/PD proteotoxicity models, significantly reducing Aβ and α-synuclein proteotoxicity, in concordance with an induced MT expression.

## Materials and Methods

### Molecular Target Prediction

The ChEMBL database version 24 was downloaded, and the chemical structures were prepared following the protocol previously reported (Mathai and Kirchmair, 2020). Compounds **1** and **2** were prepared accordingly. Next, 3D conformer ensembles were generated with OMEGA version 3.0.1.2 (all settings kept default) (Hawkins et al., 2010), and screening was performed with ROCS version 3.3.0 using the TanimotoCombo similarity measure (all settings kept default) (Hawkins et al., 2007).

### Chemicals and Reagents

The alkaloid fraction PNAF was obtained from the leaves of *P. nemorosa* as previously described (Voucher number FURB41750—Dr. Roberto Miguel Klein Herbarium; SisGen/Brazil code AA26CBC) (Klein-Júnior et al., 2020). For nemorosine A (**1**), and fargesine (**2**), isolation and elucidation procedures were described before (Klein-Júnior et al., 2020). Both compounds were dissolved in dimethyl sulfoxide (DMSO) as vehicle and stored at –20°C until use. 5-Aminoimidazole-4-carboxamide ribonucleotide (AICAR, A9978) and fluoxetine (F-132) were obtained from Sigma Aldrich with a purity ≥98%. All other substances were obtained from Merck KGaA, Darmstadt, Germany.

### Human 5-HT_2A_ Agonistic and Antagonistic Effects

The agonistic and antagonistic effects of **1** and **2** were evaluated by functional assays on 5-HT_2*A*_ receptors using HEK-293 cells with stably transfected human 5-HT_2A_. The assays were provided by Eurofins Cerep, using the protocols described previously (Porter et al., 1999). In this assay, the homogenous time-resolved fluorescence (HTRF) technology is used to measure the intracellular calcium concentration rise due to receptor activation. When 5-HT_2A_ receptors are activated, phospholipase C (PLC) hydrolyzes PIP2 and produces IP3, which binds to its receptors in the endoplasmic reticulum and releases calcium into the cytoplasm. Compounds were evaluated at 0.01, 1.0, and 10 μM, using serotonin (for agonistic) or ketanserin (for antagonistic) as positive controls. All data were normalized to the positive control wells, which were expressed as 100% signal. Mean activation of each compound concentration was compared with vehicle control with ANOVA and Bonferroni *post hoc* test.

### Molecular Docking

The two X-ray 5-HT_2A_ receptor complexes (6WGT, 6WH4) were controlled with the electron density score for individual atoms (EDIA) (Meyder et al., 2017) and downloaded from the PDB. As the PDB files contained more than one 5-HT_2A_ receptor structure, the structure with the best electron density fit of the ligand/receptor pocket was retained (chain C of 6WH4, chain B of 6WGT). The protein structures were prepared with the Protein Preparation wizard, and **1** and **2** with LigPrep (both tools are part of the Schrödinger drug discovery platform; Schrödinger, NY, United States). A cubic docking grid with a side length of 10 Å was calculated, whereby the hydroxyl groups of Ser159, Ser160, Ser239, Ser242, and Tyr370 were allowed to rotate. The ligands were docked into both docking grids with Glide (Schrödinger, New York, NY, United States) high-precision mode (XP) using default settings. Ten poses per ligand were generated. Postdocking minimization was performed with a threshold of 0.5 kcal/mol difference for rejecting minimized poses. The predicted poses were manually assessed to identify the most plausible binding mode.

### *Caenorhabditis elegans* Strains, Maintenance, and Synchronization

*Caenorhabditis elegans* wild-type var. Bristol N2; SS104, glp-4(bn2ts); CL2659, dvIs770[myo-3:human Aβ 1–42 wt:3′UTR(long) + mtl-2:GFP)]; NL5901, pkIs2386[unc-54:human α-synuclein:YFP + unc-119(+)]; CL2120, dvIs14[pCL12(unc-54:human Aβ 1–42) + mtl-2:gfp], and *Escherichia coli* strain OP50 were obtained from the Caenorhabditis Genetics Center (University of Minnesota, US). Media and agar plates were prepared as described before (Pretsch et al., 2020; Zwirchmayr et al., 2020). OP50 bacteria were grown with LB medium for 8 h at 37°C; then they were harvested by centrifugation, washed twice with ddH_2_O, and suspended in S-complete medium at 100 mg/ml. Cultivation of *C. elegans* has been done according to the protocol of the CGC. All strains were grown on plates containing nematode growth medium (NGM) seeded with OP50 at 16°C as described previously (Zwirchmayr et al., 2020). Age synchronization was done by the egg prep method from the CGC. Developmentally synchronized worms were harvested at the L3 stage (CL2659) or L4 stage (CL2120, NL5901, SS104, N2). For pharyngeal pumping assays, non-gravid young adult N2 were used.

### Locomotion Assay

Locomotion assays were performed according to the protocol of Newell Stamper et al. (2018) with minor changes. L4 N2 worms were washed from agar plates; 10–20 worms were transferred to each well of a 96-well plate containing SO medium (S-complete medium supplemented with 5 mg/ml of OP50), FUdR (120 μM) and compound stocks in triplicate. After 6 days, worms were fed again by the addition of bacterial food source OP50. Vehicle control contained 1% DMSO. Locomotion was assayed at days 10 and 16 of treatment. Worms were classified into categories I, II, III, and IV. Category IV worms were dead. Category I worms were alive as observed by pharyngeal pumping and head movements, but showed no body bending or swimming. Category II worms moved upon stimulation, which was achieved by short vortexing of the plate. Category III worms were moving spontaneously, i.e., swimming and body bending. The percentage of worms per well in each category was calculated. The mean of three triplicate wells was calculated for each experiment. The mean of three parallel experiments was calculated and compared with ANOVA and Bonferroni *post hoc* test. Hereby, category B worms of all groups were compared with control, and the sum of category B and C worms was compared with control.

### Pharyngeal Pumping Assay

Young adult N2 worms (200) were incubated in 1 ml of S-complete medium together with 2.5 mg of OP50 under agitation in 2-ml sterile Eppendorf tubes. Either 1% DMSO as vehicle control, fluoxetine as positive control, or compounds **1** and **2** (100 and 250 μM) were used as treatments. After 2 h, 10 μl, with approximately two to five worms of each cohort, was pipetted on a bacteria-free agar matrix and scored. Hereby, worms were located under the microscope, and grinder movements were counted for 20 s, three times alternately for each worm. The procedure was repeated three times, so at least 10 worms per treatment were scored. The mean pumping rate per minute of all worms were calculated and compared using ANOVA and Bonferroni *post hoc* test.

### Nile Red Assay

Nile red assay was performed as previously described (Zwirchmayr et al., 2020). A synchronized culture of L4 SS104 worms was dispensed into the wells of a 96-well plate at a density of less than 10 worms per well. OP50, Nile red, and DMSO stock solutions were added to reach a final concentration of 10 mg/ml, 100 nM, and 1% in 100 μl, respectively. Vehicle control was treated with 1% DMSO. Positive control AICAR, **1** and **2** were tested at 100 μM. Worms were kept in darkness at 25°C for 4 days, controlled, and paralyzed with NaN_3_ prior to imaging using a Zeiss Axio Observer Z1 inverted fluorescence microscope (Carl Zeiss, Vienna, Austria) equipped with a rhodamine filter and an Axio Cam MRm camera system. Every worm was recorded using the same settings and same subsaturating exposure times. The open source software ImageJ was used for image processing and quantification of fluorescence as previously described (Lehner et al., 2021). The experiment was repeated at least three times with six wells per treatment. Fluorescence was expressed as % fluorescence, whereby the vehicle control worm fluorescence was set to 100%. The mean worm fluorescence of each treatment was compared with ANOVA and Bonferroni *post hoc* test.

### Aβ Accumulation Assay

The strain CL2659 was used, where Aβ expression is induced by temperature upshift from 16°C to 25°C, which results in a paralysis phenotype within approximately 48 h in liquid culture. Treatments that inhibit Aβ toxicity in this model alter the rate of paralysis in these worms. The assay was performed as described before (Pretsch et al., 2020). Synchronized L3 CL2659 larvae (10–20) were added to each well of a 96-well in SO medium (S-complete medium supplemented with 5 mg/ml of OP50). Stock solutions were added to reach a final concentration of 1% DMSO in 100 μl per well. Each treatment was tested in triplicate. Aβ transgene expression in muscle cells was induced by temperature upshift. On day 0 (before temperature upshift) and day 2 (48 h after temperature upshift), the number of paralyzed worms was scored under a dissecting microscope. For generation of bar graphs, the mean ratio of paralyzed worms was given as percentage at days 0 and 2. The mean paralysis rate of each treatment was compared with control using one-way ANOVA and Bonferroni *post hoc* test.

### Metallothionein and α-Synuclein Assays

The MT assay was performed with strain CL2120 and the α-synuclein misfolding assay with strain NL5901 according to the previously described protocols (Pretsch et al., 2020). In brief, 90 μl of worm suspension containing L4 larvae in SO medium at a concentration of about 50 worms/well was added to each well of a 96-well plate. Ten-microliter compound dilutions were added in triplicate in given concentrations. MT expression was followed by measuring GFP at days 0, 3, and 6; α-synuclein expression was followed by measuring YFP at days 0, 3, and 6 with a Tecan Infinite 200 pro (Tecan AG, Männedorf, Switzerland) at 450/535 nm. Fluorescence of vehicle control (α-synuclein) or positive control (MT assay) was set to 100% fluorescence change. The difference in fluorescence increase or decrease between compound-treated wells and vehicle control on two different days was analyzed using one-way ANOVA and Bonferroni *post hoc* test, and a two-tailed Student’s *t*-test, respectively (*n* = 30–50/well).

## Results and Discussion

### *Psychotria* Alkaloids Are Activators of Human 5-HT_2A_ Receptor

To identify the putative targets of **1** and **2**, the alkaloids of interest were compared with a large set of compounds with measured bioactivities for a total of 4,600 proteins. This bioactivity data set is a subset of the ChEMBL database (Davies et al., 2015; Mendez et al., 2019) that was compiled and curated by some of us previously (Mathai and Kirchmair, 2020). The screening engine ROCS (Hawkins et al., 2007) was employed for pairwise comparisons of aligned, 3D molecular shapes, taking also the alignment of chemical features such as hydrogen bond donors, hydrogen bond acceptors, and hydrophobic moieties into account. ROCS has previously been shown to perform well even in cases where the compounds of interest are only remotely related to compounds in the reference set (Chen et al., 2020).

By screening **1** and **2** against the reference set, a rank-ordered list of the 4,600 proteins covered by a bioactivity data set was obtained. The top 20 targets are reported in the Supplementary Tables 1,2. Intriguingly, for each compound, 14 out of the top 20 predicted targets are known to be of significance to the treatment of NDs. These targets include indolamine-2,3-dioxygenase (IDO), poly(ADP-ribose)polymerase-1 (PARP1), and 5-hydroxytryptamine receptors (5-HTR). Moreover, the two known targets of **1** and **2**, MAO-A and BuChE, were assigned lower ranks, but still among the top 150. More specifically, MAO-A was ranked 90 and 45 for **1** and **2**, and BuChE 105 and 137, respectively. The lower ranks of these targets can be explained by the fact that all the active compounds for these targets recorded in the reference set (and even in the unprocessed, complete ChEMBL database) are structurally clearly dissimilar from **1** and **2**.

**FIGURE 2:**
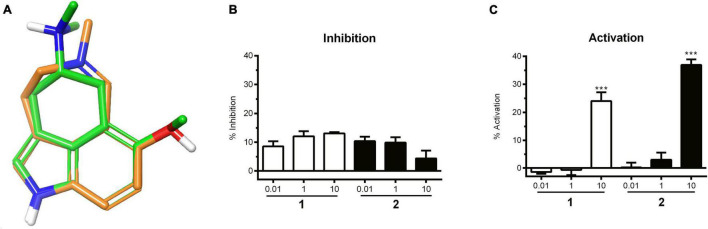
**(A)** Nemorosine A (**1**) aligned with 6-methoxy-*N,N*-dimethyl-1,3,4,5-tetrahydrobenzo[*cd*]indol-4-amine (ChEMBL49309), a known antagonist of the 5-HT_1_ and 5-HT_2_ receptors. **(B)** 5-HT competitive inhibition of 5HT_2A_ by **1** and **2** at different concentrations in HEK-293T cells. Data were normalized to the positive control wells (ketanserin), which were expressed as 100% inhibition. Bars represent the mean inhibition of two independent experiments ± SD. **(C)** Concentration-dependent increase in 5HT_2A_ activity in HEK-293T cells in response to **1** and **2**. Bars represent the mean of two independent experiments ± SD. Vehicle control was set to 0% inhibition. Significance was evaluated with one-way ANOVA–Bonferoni *post hoc* test (***p < 0.001 vs. vehicle control).

The prediction of the 5-HTR interaction was considered particularly promising to us, given the good quality of 3D alignment of the alkaloids to known modulators of the 5-HT_2_ receptor, such as 6-methoxy-*N,N*-dimethyl-1,3,4,5-tetrahydrobenzo[*cd*]indol-4-amine (ChEMBL49309) (Figure 2A). ChEMBL49309 has been reported as an antagonist on rat 5-HT_1_ and 5-HT_2_ receptors (Flaugh et al., 1988). Accordingly, the isolated alkaloids were subjected to experimental evaluation on the (human) 5-HT_2A_ receptor in cellular functional assays.

Agonistic and antagonistic effects were evaluated for nemorosine A (**1**) and fargesine (**2**) using functional assays on 5-HT_2A_ receptors stably transfected in HEK-293 cells. No dose-dependent antagonistic effect was observed for the isolated alkaloids (Figure 2B). On the other hand, **1** and **2** (at 10 μM) exhibited agonistic effects on the 5-HT_2A_ receptor in the order of 24.0 ± 3.2% and 37.0 ± 2.0% of serotonin response, respectively (Figure 2C). The results demonstrated that both **1** and **2** are able to activate 5-HT_2A_ receptors to some extent. 5-HT_2A_ is widely distributed in the brain, and agonists are related to cognitive-enhancing and hallucinogenic activities. 5-HT_2A_ agonists, such as psilocybin, have drawn interest for their anxiolytic and antidepressant effects. There is increasing evidence that 5-HT_2A_ receptor agonists modulate learning and memory function by improving neuroplasticity, functional neuronal connectivity, and activating anti-inflammatory pathways (de Vos et al., 2021; Garcia-Romeu et al., 2021; Kozlowska et al., 2021). It was also demonstrated that the neocortical 5-HT_2A_ receptor binding is decreased in patients with AD (Hasselbalch et al., 2008), which is correlated to cognitive impairment. Thus, 5-HT_2A_ activators came into focus as potential pharmacotherapies for patients with AD and other dementias (Vann Jones and O’Kelly, 2020).

**FIGURE 3:**
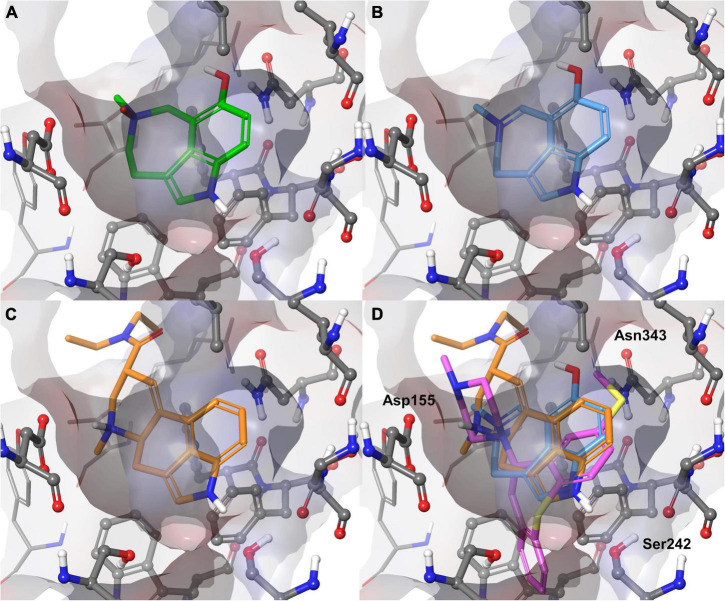
Binding poses predicted for **1** (**A**, green) and **2** (**B**, blue) with the 5-HT_2A_ receptor compared with the experimental complex of the 5-HT_2A_ receptor with the agonist LSD (**C**, orange) and superimposition of **2** with LSD (orange) and inverse agonist methiothepin (purple) **(D)**. Three crucial side chains (Asp155, Ser242, Asn343) for interactions with the alkaloids are labeled. Val156 would have covered the bag from the shown perspective and was blanked for better visibility.

To gain molecular insight into the binding mode of **1** and **2** with the 5-HT_2A_ receptor, molecular docking was performed. Two X-ray crystal structures, complexed with the activator lysergic acid diethylamide (LSD) and the inverse agonist methiothepin (Kim et al., 2020) were used. Alkaloids **1** and **2** were predicted to bind very similar to LSD. Their poses can be aligned very well to the ergoline structure (Figure 3). Hereby, the positively charged azepine nitrogen forms a salt bridge with Asp155, which is essential for the activation of monoamine receptors (Kristiansen et al., 2000). Further hydrophobic interactions are predicted to be formed with Val156, Leu229, Phe234, Phe339, and Phe340. The indole N-H of **1** and **2** is predicted to form a hydrogen bond with Ser242, similar to the one observed in the LSD complex (Kim et al., 2020). Additionally, a hydrogen bond between the 7-OH with Asn343 was predicted. According to molecular docking and mutagenesis studies, this interaction is crucial for receptor activation by 5-HT (Kristiansen et al., 2000; Kim et al., 2020). The docking poses obtained for **1** and **2** are plausible and in agreement with many studies on 5-HT_2A_ receptor agonism (Kristiansen et al., 2000; Braden and Nichols, 2007; Kim et al., 2020).

### Phenotypic Effects in Models of *Caenorhabditis elegans*

**FIGURE 4:**
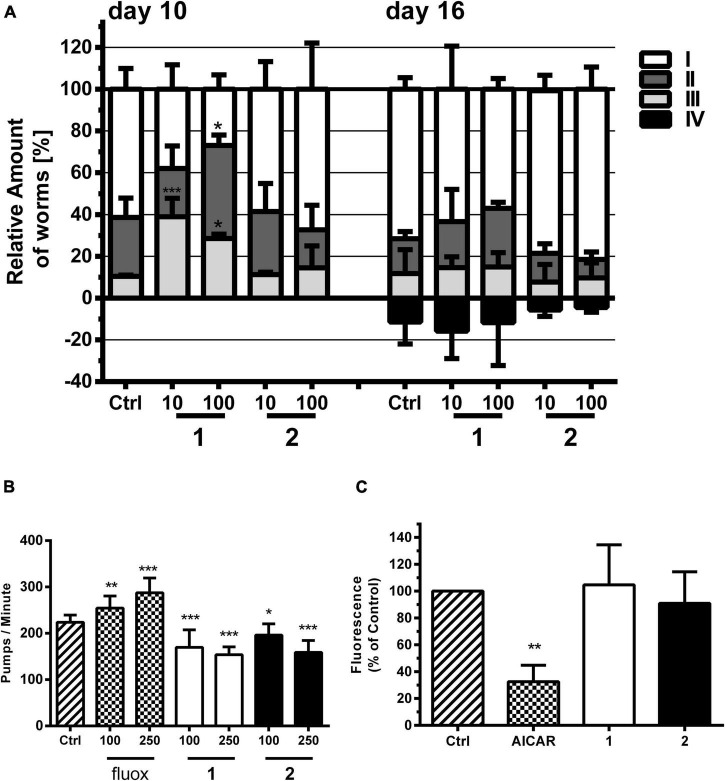
Effects of alkaloids **1** and **2** on locomotion, pharyngeal pumping, and fat accumulation of *C. elegans.* **(A) 1** but not **2** stimulated locomotion compared with vehicle control (Ctrl, 1% DMSO). **1** and **2** were tested at 10 and 100 μM. Locomotion was analyzed by movement observations after short vortexing of the plate at days 10 and 16. The individuals were classified according to the movement: I—alive but no movements, II—spontaneous movement, III—movement after stimulus, IV—dead. Bars represent the mean of three parallel experiments ± SD. Significance was evaluated with one-way ANOVA and Bonferroni *post hoc* test (vs. Ctrl; *p < 0.05; ***p < 0.001). **(B)** Effects of pharyngeal pumping of N2 worms by **1** and **2** and the 5-HT reuptake inhibitor fluoxetine (fluox) in comparison with vehicle control (Ctrl, 1% DMSO). Young adult N2 hermaphrodites were treated for 2 h with bacteria and then transferred on an agar matrix. Pumps based on grinder movements were counted with a stereomicroscope. Bars represent the mean of 10–20 animals ± SD. Significance was evaluated with one-way ANOVA and Bonferroni *post hoc* test (vs. Ctrl; *p < 0.05; **p < 0.01; ***p < 0.001). **(C)** Differences in lipid-derived Nile red fluorescence of *C. elegans* strain CL2659 treated with vehicle control (Ctrl, 1% DMSO), positive control 5-aminoimidazole-4-carboxamide ribonucleotide (AICAR), and alkaloids **1** and **2** (each 100 μM). Bars represent the mean fluorescence intensities of three independent experiments expressed as % of control worms ± SD. Significance was assessed by one-way ANOVA and Bonferroni *post hoc* test (**p < 0.01).

In exploring the 5-HT-related activities of **1** and **2** in the model organism *C. elegans*, relevant drug behavioral assays were performed, i.e., pharyngeal pumping, locomotion, and fat accumulation of worms (Figure 4). 5-HT is a conserved neuromodulator in mammals and nematodes. Thus, the tiny roundworm *C. elegans* represents an opportunity to probe our multitarget ligands in an *in vivo* model at the scale of a cell-based assay. Exogenous 5-HT generates stereotypical phenotypes in *C. elegans*, which are mediated by a serotoninergic system of high similarity to mammals. Exogenous 5-HT simulates a “food is available”-like signal, which results in (i) reduced locomotion, (ii) increased feeding, and (iii) increased energy utilization and egg laying (Hapiak et al., 2009). Several 5-HT-sensitive GPCRs are known from *C. elegans*: SER-1 is likely coupled to a G_αq_ protein and a Ca^2+^-mediated signaling pathway and resembles 5-HTR_2_ (Hamdan et al., 1999; Bastiani et al., 2003). SER-4 attenuates and SER-7 stimulates adenylate cyclase activity upon serotonin binding, resembling 5-HTR_1_ and 5-HTR_7_, respectively (Olde and McCombie, 1997; Carre-Pierrat et al., 2006).

We tested the effect of **1** and **2** on the locomotion of N2 *C. elegans* with an assay similar to that of Newell Stamper et al. (2018), whereby worms were classified into the categories spontaneously moving (swimming and body bending) worms, worms which only move upon stimulation, and not moving animals (besides pharyngeal pumping and some head and tail movements). The assay was performed at a concentration of 10 and 100 μM of **1** and **2** and at two different timepoints in liquid medium. We could observe that the locomotion of worms was significantly higher upon treatment with **1**, but not with **2** (Figure 4A). This is in contrast to the effect described for a SER-1 receptor agonist, which inhibits locomotion, e.g., fluoxetine (Dernovici et al., 2007). One may speculate that **1** and **2** protect the worms from an age-dependent movement decline. Similar to other animals, aging nematodes experience sarcopenia and loss of neuromuscular function (Herndon et al., 2002).

In line with the results from the locomotion assay, **1** and **2** do not induce, but inhibit, pharyngeal pumping of worms (Figure 4B). *Caenorhabditis elegans* uses its pharynx, a neuromuscular pump, to grind and ingest bacteria into its intestine. The pumping is stimulated either by the presence of a bacterial food source or by exogenous 5-HT. The GPCRs SER-1, SER-4, and SER-7 are essential as their null allele mutants fail to produce a fast pumping behavior (Hobson et al., 2006; Lee et al., 2017). 5-HT and other agonists of the 5-HT-regulated GPCRs, as well as 5-HT reuptake inhibitors (Scabia et al., 2018), such as fluoxetine, increase pharyngeal pumping (Almotayri et al., 2021). Antagonists of these proteins are reported to inhibit pharyngeal pumping (Niacaris and Avery, 2003) similar to what is observed in this study for **1** and **2**.

5-HT reuptake inhibitors, such as fluoxetine, usually result in a low-fat phenotype by stimulation of mitochondrial beta-oxidation (Srinivasan et al., 2008). The neuronal circuit underlying this effect is poorly understood, but requires the 5-HT-sensitive SER-6 receptor as well as the 5-HT-regulated chloride channel MOD-1 (Noble et al., 2013). Similar to the other phenotypic assays, **1** and **2** showed no 5-HT-related effect (Figure 4C). SS104 mutant worms treated with **1** and **2** for 4 days resulted in a similar body fat content to the vehicle control-treated animals using a Nile red staining assay established by some of us previously (Zwirchmayr et al., 2020).

Summarized, the behavioral profiles of **1** and **2** in *C. elegans* with increased locomotion, reduced pharyngeal pumping, and a normal body fat content point out that at least in nematodes, they rather act as 5-HT receptor antagonists than agonists. One may speculate about the different effects observed *in vitro* (on the human 5-HT receptor) and *in vivo*. Possible explanations are (i) differences in human 5-HT_2A_ and nematode SER-1 receptors, (ii) cholinergic activities prevailing over the serotoninergic activation, or (iii) yet undiscovered mechanisms. It has to be stressed that **1** and **2** are cholinesterase inhibitors (Klein-Júnior et al., 2020). Therefore, it is expected that the multifunctional profile of these alkaloids may play different roles in several phenotypic behaviors. Locomotion, for instance, is controlled by acetylcholine, promoting excitation of body wall muscles. The same effect is observed for pharyngeal pumping, which is excited by cholinergic inputs (Treinin and Jin, 2021).

Interestingly, the modulation of serotonergic signaling as observed in the used assays has been shown to protect against protein toxicity in *C. elegans* (Teixeira-Castro et al., 2015). The *in silico* predicted and *in vitro* experimentally confirmed target profile strengthened the hypothesis that **1** and **2** could have a protective effect against protein toxicity. This prompted us to test the two alkaloids in assay-related protein toxicity models of *C. elegans*.

### *Caenorhabditis elegans* Protection From Protein Toxicity

**FIGURE 5:**
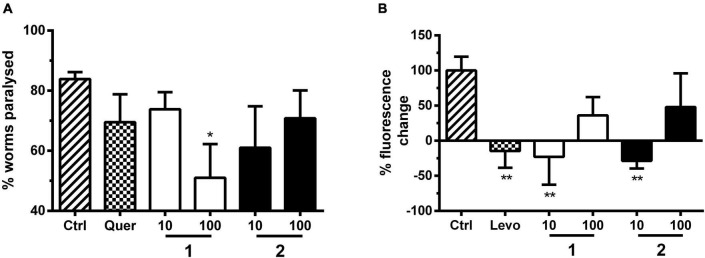
**(A)** Paralysis assay with *C. elegans* strain CL2659. Bars represent % paralyzed worms in the vehicle control group [Ctrl, 1% (DMSO)], positive control group (quer, quercetin; 10 μM) and treatment groups (**1** and **2**; 10 and 100 μM) 48 h after Aβ induction, as mean ± SD of three parallel experiments. Significance was assessed with one-way ANOVA and Bonferroni *post hoc* test (*p < 0.05 vs. Ctrl). **(B)** Yellow fluorescent protein (YFP)-tagged α-synuclein expression assay with *C. elegans* strain NL5901. Bars represent the mean worm fluorescence change ± SD between day 3 and day 6 of treatment measured in three parallel experiments. Vehicle control group (Ctrl) was treated with 1% DMSO, positive control group with levodopa (Levo, 2 mM), and treatment groups with **1** and **2** (10 and 100 μM). Fluorescence change of vehicle control group was set to 100% fluorescence change. Significance was assessed with one-way ANOVA and Bonferroni *post hoc* test (**p < 0.01 vs. Ctrl).

**1** and **2** were tested with a *C. elegans* paralysis assay using strain CL2659 (Figure 5A). Unlike the human amyloid precursor protein (APP) gene, the *C. elegans* homolog gene apl-1 cannot produce the neurotoxic peptide Aβ. The strain CL2659 has been engineered by Fonte et al. (2011) to inducibly express Aβ upon temperature upshift in muscle cells, which causes a paralyzed phenotype within 48 h. Treatments that counter Aβ toxicity in such models, e.g., by treatment with *Ginkgo biloba* extract (Wu et al., 2006) or supplementation of dietary vitamin B12 (Lam et al., 2021), reduce the rate of paralysis in these worms. In our experiments, the two alkaloids **1** and **2** protected against Aβ proteotoxicity observed by a reduced paralysis rate of treated worms compared with control. The % reduction in paralyzed worms treated with **1** and **2** at each concentration was similar to our positive control quercetin (10 μM) (Figure 5A and Supplementary Table 3). Alkaloid **1** at 100 μM even halved the number of paralyzed worms.

We further evaluated the effect of these alkaloids on *C. elegans* transgenic strain NL5901 endowed with YFP-tagged α-synuclein (Figure 5B). The transgenic NL5901 strain allows the direct monitoring of α-synuclein inclusion formation *via* YFP fluorescence in living worms (van Ham et al., 2008). This proteotoxicity model mimics human PD synucleopathies with dopaminergic neuronal loss and motor deficits (Lakso et al., 2003). Similar to the positive control levodopa at 2 mM, the two alkaloids **1** and **2** at 10 μM significantly reduced the α-synuclein toxicity after 6 days of treatment (Figure 5B and Supplementary Table 3).

**FIGURE 6:**
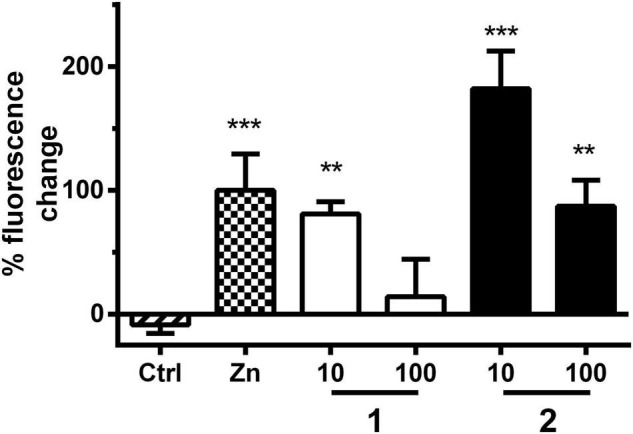
MT assay with strain CL2120. Bars represent the mean green fluorescent protein (GFP)-derived fluorescence change ± SD between day 3 and day 6 derived from MT expression in worms treated either with vehicle control (Ctrl, 1% DMSO), positive control (Zn, ZnSO_4_, 10 μM), or treatment with **1** or **2** (10 and 100 μM). Positive control fluorescence increase was set to 100% fluorescence change. Significance was assessed with one-way ANOVA and Bonferroni *post hoc* test (**p < 0.01; ***p < 0.001 vs. Ctrl).

The stimulation of serotonin receptors has previously been reported to play an important role in astrocytes by the upregulation of MTs, leading to a neuroprotective effect (Isooka et al., 2020; Kikuoka et al., 2020). Sequeira et al. (2012) observed a correlation between a decreased expression of both 5-HT_2A_ receptors and MTs in mood disorder patients. MTs are divided into four isoforms (Zarêba and Kepinska, 2020). These proteins play an important role in the transport, storage, and homeostasis of some essential metal ions, such as Zn^2+^ and Cu^2+^, as well as the detoxification of heavy metals (Koh and Lee, 2020; Pretsch et al., 2020; Zarêba and Kepinska, 2020). It has been observed that MT-1 and MT-2, protective factors against neuronal damage and ROS, are overexpressed in patients with AD (Zarêba and Kepinska, 2020). On the other hand, MT-3 expression is controversial (Atrián-Blasco et al., 2017; Koh and Lee, 2020; Pretsch et al., 2020; Zarêba and Kepinska, 2020). MT-3 also exerts important protective effects. However, this isoform seems to play a more complex role, since it is also involved, for example, in Aβ endocytosis in the astrocytes by modulating actin polymerization (Koh and Lee, 2020; Zarêba and Kepinska, 2020). Recently, Pretsch et al. (2020) demonstrated that the prolongation of MT induction time is correlated to a reduction in Aβ toxicity in *C. elegans*. They observed that in Aβ-expressing worms, MT is overexpressed in young adults, but its levels are largely reduced during aging. On the other hand, healthy worms, not expressing Aβ, show a discrete increase in MT levels with age. Therefore, the role of MT in the positive results derived from the paralysis assay was investigated, using the *C. elegans* transgenic strain CL2120, where MT is GFP tagged, and Aβ is expressed constitutively. The treatment with **1** and **2** (at 10 and 100 μM) significantly prolonged MT induction time in *C. elegans*, which is in line with a reduced Aβ proteotoxicity (Figure 6 and Supplementary Table 3), similar to the results obtained previously for quercetin, apomorphine, and other known MT inducers (Pretsch et al., 2020). MTs also play a neuroprotective role in PD (Isooka et al., 2020; Kikuoka et al., 2020). It was demonstrated that MTs are overexpressed in astrocytes and reduced in neurons of PD patients (Juárez-Rebollar et al., 2017; Okita et al., 2017). In addition to the antioxidant role, MTs modulate the bioavailability of metals, such as Cu^2+^, which accumulates in the brain during aging and binds to α-synuclein and aggregating as well as originating Lewy bodies (Okita et al., 2017). These are markers of PD, localized mainly in dopaminergic neurons, hampering dopamine transmission (Murakami et al., 2014). Therefore, increasing the expression of MTs is hypothesized as a possible mechanism of action of disease-modifying therapies (Okita et al., 2017). Recently, Pretsch et al. corroborated this hypothesis by demonstrating that the prolongation of MT induction reduces α-synuclein toxicity in *C. elegans* (Pretsch et al., 2020).

## Conclusion

To summarize, in this study, a combined *in silico*, *in vitro*, and *in vivo* approach was performed to assess the impact of the two main alkaloids of *P. nemorosa*, namely, nemorosine A (**1**), and fargesine (**2**) on protein toxicity-related NDs. The data obtained from the different nematode models and the target-based assays corroborate the multifunctional profile of these azepine-indole alkaloids both in AD and PD. They modulate symptomatic and disease-modifying targets. How particularly 5-HT receptor modulation contribute to the observed effects against protein toxicity is an important question, which in the future should be answered by *in vitro* cell culture experiments. Finally, the identification of molecular mechanism of NPs against amyloid/α-synuclein toxicities might be key to develop urgently needed therapeutic solutions against ND (Pagano et al., 2020). The results of this study underline the potential therapeutic effects of the investigated *Psychotria* species and reinforce the importance of *Psychotria* genus as a promising source of chemical entities with new scaffolds for the search of bioactive compounds on targets related to NDs.

## Data Availability Statement

The original contributions presented in the study are included in the article/Supplementary Material, further inquiries can be directed to the corresponding author.

## Author Contributions

BK, LK-J, and JR contributed to the conceptualization. YC and JK performed *in silico* target prediction experiments. BK and LK-J reviewed the predicted targets and initiated 5-HT_2A_ testing. LK-J and SC dealt with *in vitro* assays and provided the alkaloids. BK performed the protein-ligand docking experiments and the Nile red, pharyngeal pumping assays, and prepared the figures. DP performed the Locomotion, Aβ and α-synuclein accumulation as well as MT expression assays. LK-J, BK, and DP performed the interpretation of results and wrote the manuscript. AG, YH, PC, JK, AH, and JR supported the study. All authors reviewed and edited the manuscript.

## Conflict of Interest

The authors declare that the research was conducted in the absence of any commercial or financial relationships that could be construed as a potential conflict of interest.

## Publisher’s Note

All claims expressed in this article are solely those of the authors and do not necessarily represent those of their affiliated organizations, or those of the publisher, the editors and the reviewers. Any product that may be evaluated in this article, or claim that may be made by its manufacturer, is not guaranteed or endorsed by the publisher.
