# Supplementary material for: Azepine-Indole Alkaloids From Psychotria nemorosa Modulate 5-HT2A Receptors and Prevent in vivo Protein Toxicity in Transgenic Caenorhabditis elegans
Source: Front Neurosci. 2022 Mar 14;16:826289. doi: 10.3389/fnins.2022.826289 (PMC8963987; doi:10.3389/fnins.2022.826289)
Supplement: Supplementary file 1 [file Data_Sheet_1.pdf]

Supplementary Material

Table S1. Top 20 ranked targets for nemorosine A (**1**).

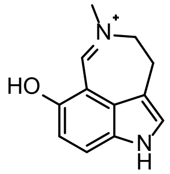	**Chembl ID**	**TC score (1)**	**Target**	**Role**	**Significance in neurodegenerative diseases**
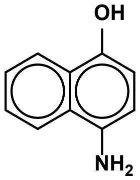	CHEMBL576321	1,60	IDO<br><br>CHEMBL4685	tryptophan-metabolism	[1–3]
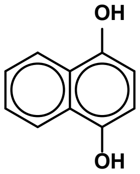	CHEMBL206816	1,59	IDO1<br><br>CHEMBL1075294	tryptophan-metabolism	
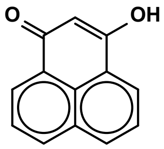	CHEMBL1446983	1,54	female germline-specific tumor suppressor gld-1<br><br>CHEMBL1293302	entry into meiosis<br><br>(*C. elegans*)	-
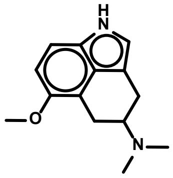	CHEMBL49309	1,52	5-HTR 2<br><br>CHEMBL2093870	serotonergic system	[4–7]
			5-HTR 1<br><br>CHEMBL2095159	serotonergic system	

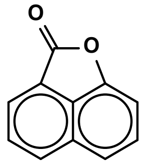	CHEMBL2323363	1,52	thymidylate synthase<br><br>CHEMBL1952	DNA<br><br>biosynthesis	-
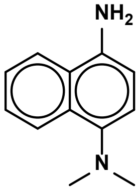	CHEMBL1998253	1,51	DYRK1A<br><br>CHEMBL2292	cell cycle control<br><br>cell differentiation	[8]
			NEK2<br><br>CHEMBL3835	cell cycle control	-
			LIMK1<br><br>CHEMBL3836	cytoskeleton architecture	-
			CLK4<br><br>CHEMBL1998253	spliceosome regulation	-
			CHECK1<br><br>CHEMBL4630	DNA damage response<br><br>cell cycle control	-
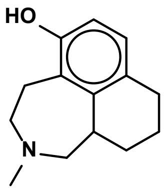	CHEMBL1203602	1,51	adrenergic receptor α2<br><br>CHEMBL2093864	norepinephrine system	[9]
			5-HT1aR<br><br>CHEMBL273	serotonergic system	[4–7]

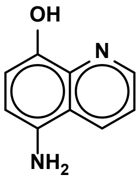	CHEMBL449298	1,51	TDO<br>CHEMBL1075307	tryptophan-metabolism	[10,11]
			HSP90<br>CHEMBL2095165	chaperone	[12]
			proteasome macropain subunit MB1<br>CHEMBL4662	protein degradation	[13]
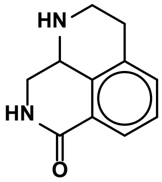	CHEMBL608994	1,50	PARP1<br>CHEMBL3105	DNA repair	[14–16]
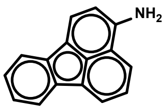	CHEMBL103583	1,48	CRF-BP<br>CHEMBL3885546	stress response	[17,18]
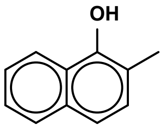	CHEMBL122451	1.48	COX<br>CHEMBL2096674	inflammation	[19,20]
		1,48	5-LO<br>CHEMBL312	inflammation	[21,22]

Table S2. Top 20 ranked targets for fargesine (**2**).

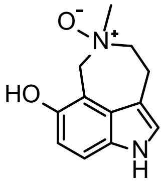	**Chembl ID**	**TC score (2)**	**Target**	**Role**	**Significance in neurodegenerative diseases**
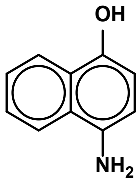	CHEMBL576321	1,47	IDO<br>CHEMBL4685	tryptophan-metabolism	[1–3]
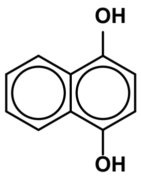	CHEMBL206816	1,47	IDO1<br>CHEMBL1075294	tryptophan-metabolism	
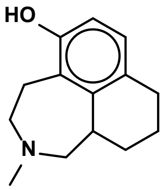	CHEMBL1203602	1,44	adrenergic receptor α2<br>CHEMBL2093864	norepinephrine system	[9]
			5-HT1aR<br>CHEMBL273	serotonergic system	[4–7]
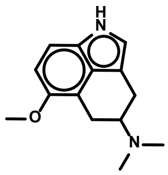	CHEMBL49309	1,44	5-HTR 2<br>CHEMBL2093870	serotonergic system	[4–7]
			5-HTR 1<br>CHEMBL2095159	serotonergic system	

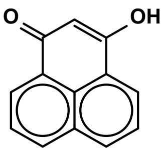	CHEMBL1446983	1,41	female germline-specific tumor suppressor gld-1<br><br>CHEMBL1293302	entry into meiosis<br><br>(*C. elegans*)	-
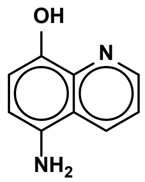	CHEMBL449298	1,40	TDO<br><br>CHEMBL1075307	tryptophan-metabolism	[10,11]
			HSP90<br><br>CHEMBL2095165	chaperone	[12]
			proteasome macropain subunit MB1<br><br>CHEMBL4662	protein degradation	[13]
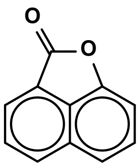	CHEMBL2323363	1,38	thymidylate synthase<br><br>CHEMBL1952	DNA<br><br>biosynthesis	-
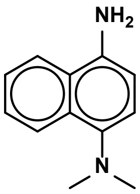	CHEMBL1998253	1.38	DYRK1A<br><br>CHEMBL2292	cell cycle control<br><br>cell differentiation	[8]
			NEK2<br><br>CHEMBL3835	cell cycle control	-
			LIMK1<br><br>CHEMBL3836	cytoskeleton architecture	-
			CLK4<br><br>CHEMBL1998253	spliceosome regulation	-
			CHECK1<br><br>CHEMBL4630	DNA damage response<br><br>cell cycle control	-

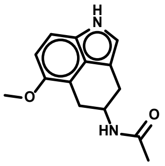	CHEMBL61418	1.38	melatonin receptor<br>CHEMBL2094268	entraining sleep/wake	[7,23]
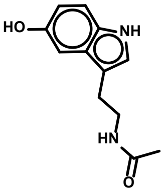	CHEMBL33103	1,37	melatonin receptor<br>CHEMBL2095154	entraining sleep/wake	[7,23]
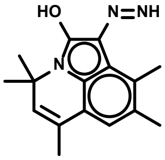	CHEMBL1328384	1,37	streptokinase A	thrombolytic medication	-
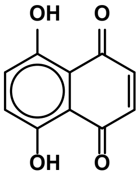	CHEMBL274056	1,37	5-LO<br>CHEMBL2980	inflammation	[21,22]
			CDC25B<br>CHEMBL4804	cell cycle control	-
			DUSP6<br>CHEMBL5511	negative feedback of MAPK pathway	[24]
			DUSP1<br>CHEMBL5623	negative feedback of MAPK pathway	

Abbreviations.

5-HTR, 5-hydroxytryptamine receptor; 5-LO, arachidonate 5-lipoxygenase; CHECK1, serine/threonine-protein kinase Chk1, CDC25B, dual specificity phosphatase CDC25B; CLK4, dual specificity protein kinase CLK4; COX, cyclooxygenase; CRF-BP, corticotropin-releasing factor receptor 2/corticotropin-releasing factor-binding protein; DUSP1, Dual specificity protein phosphatase 1; DUSP6 Dual specificity protein phosphatase 6; DYRK1A, dual-specificity tyrosine-phosphorylation regulated kinase 1A; HSP90, heat shock protein 90; IDO, indolamin-2,3-dioxygenase; LIMK1, LIM domain kinase 1; NEK2, serine/threonine-protein kinase NEK2; PARP1, poly[ADP-ribose]polymerase-1; TC, Tanimoto combo; TDO, tryptophan 2,3-dioxygenase

References

1. Bonda, D. J. *et al.* Indoleamine 2,3-dioxygenase and 3-hydroxykynurenine modifications are found in the neuropathology of Alzheimer's disease. *Redox Rep.* **15**, 161–168 (2010).

2. Maramai, S., Benchekroun, M., Gabr, M. T. & Yahiaoui, S. Multitarget therapeutic strategies for Alzheimer's disease: Review on emerging target combinations. *Biomed Res. Int.* **2020**, ID 5120230 (2020).

3. Campbell, B. M., Charych, E., Lee, A. W. & Möller, T. Kynurenines in CNS disease: Regulation by inflammatory cytokines. *Front. Neurosci.* **8**, 1–22 (2014).

4. Sierksma, A. S. R., van den Hove, D. L. A., Steinbusch, H. W. M. & Prickaerts, J. Major depression, cognitive dysfunction and Alzheimer's disease: Is there a link? *Eur. J. Pharmacol.* **626**, 72–82 (2010).

5. Isooka, N. *et al.* Dopaminergic neuroprotective effects of rotigotine via 5-HT_{1A} receptors: Possibly involvement of metallothionein expression in astrocytes. *Neurochem. Int.* **132**, 104608 (2020).

6. Kikuoka, R. *et al.* Mirtazapine exerts astrocyte-mediated dopaminergic neuroprotection. *Sci. Rep.* **10**, 1–15 (2020).

7. Herrera-Arozamena, C., Martí-Marí, O., Estrada, M., De La Revenga, M. F. & Rodríguez-Franco, M. I. Recent advances in neurogenic small molecules as innovative treatments for neurodegenerative diseases. *Molecules* **21**, 1165 (2016).

8. Jarhad, D. B., Mashelkar, K. K., Kim, H. R., Noh, M. & Jeong, L. S. Dual-specificity tyrosine phosphorylation-regulated kinase 1A (DYRK1A) inhibitors as potential therapeutics. *J. Med. Chem.* **61**, 9791–9810 (2018).

9. Tatton, W., Chen, D., Redman, R. C., Wheeler, L., Nixon, R. & Tatton N. Hypothesis for a common basis for neuroprotection in glaucoma and Alzheimer's disease: Anti-apoptosis by alpha-2-adrenergic receptor activation. *Surv. Ophthalmol.* **48**, S25-37 (2003).

10. Van der Goot, A. T., Zhu, W., Vázquez-Manrique, R. P., Seinstra, R. I., Dettmer, K., Michels, H., Farina, F., Krijnen, J., Melki, R., Buijsman, R. C., Silva, M. R., Thijssen, K. L., Kema, I. P., Neri, C., Oefner, P. J. & Nollen, E. A. A. Delaying aging and the aging-associated decline in protein homeostasis by inhibition of tryptophan degradation. *Proc. Natl. Acad. Sci. U. S. A.* **109**,

14912–14917 (2012).

11. Sorgdrager, F. J. H., van Der Ley, C. P., van Faassen, M., Calus, E., Nollen, E. A., Kema, I. P., van Dam, D. & De Deyn, P. P. The effect of tryptophan 2,3-dioxygenase inhibition on kynurenine metabolism and cognitive function in the APP23 mouse model of Alzheimer's disease. *Int. J. Tryptophan Res.* **13**, 1178646920972657 (2020).

12. Pratt, W. B., Gestwicki, J. E., Osawa, Y. & Lieberman, A. P. Targeting Hsp90/Hsp70-based protein quality control for treatment of adult onset neurodegenerative diseases. *Annu. Rev. Pharmacol. Toxicol.* **55**, 353–371 (2015).

13. Bi, M., Du, X., Jiao, Q., Chen, X. & Jiang, H. Expanding the role of proteasome homeostasis in Parkinson's disease: Beyond protein breakdown. *Cell Death Dis.* **12**, 154 (2021).

14. Mao, K. & Zhang, G. The role of PARP1 in neurodegenerative diseases and aging. *FEBS J.* 1–12 (2021).

15. Martire, S., Mosca, L. & Erme, M. PARP-1 involvement in neurodegeneration : A focus on Alzheimer ' s and Parkinson ' s diseases. **148**, 53–64 (2015).

16. Lee, Y., Kang, H. C., Lee, B. D., Lee, Y. I., Kim, Y. P. & Shin, J. H. Poly (ADP-ribose) in the pathogenesis of Parkinson's disease. *BMB Rep.* **47**, 424–432 (2014).

17. Kempuraj, D., Selvakumar, G. P., Thangavel, R., Ahmed, M. E., Zaheer, S., Raikwar, S. P., Iyer, S. S., Bhagavan, S. M., Beladakere-Ramaswamy, S. & Zaheer, A. Mast cell activation in brain injury, stress, and post-traumatic stress disorder and Alzheimer's disease pathogenesis. *Front. Neurosci.* **11**, 1–15 (2017).

18. Vandael, D. & Gounko, N. V. Corticotropin releasing factor-binding protein (CRF-BP) as a potential new therapeutic target in Alzheimer's disease and stress disorders. *Transl. Psychiatry* **9**, 272, (2019).

19. Teismann, P., Tieu, K., Choi, D. K., Wu, D. C., Naini, A., Hunot, S., Vila, M., Jackson-Lewis, V. & Przedborski, S. Cyclooxygenase-2 is instrumental in Parkinson's disease neurodegeneration. *Proc. Natl. Acad. Sci. U. S. A.* **100**, 5473–5478 (2003).

20. Minghetti, L. Cyclooxygenase-2 (COX-2) in inflammatory and degenerative brain diseases. *J. Neuropathol. Exp. Neurol.* **63**, 901–910 (2004).

21. Michael, J., Marschallinger, J. & Aigner, L. The leukotriene signaling pathway: a druggable target in Alzheimer's disease. *Drug Discov. Today* **24**, 505–516 (2019).

22. Siddiqui, A., Akhtar, M. S., Shah, Z., Othman, I. & Kumari, Y. Inflammation drives Alzheimer's disease: Emphasis on 5-lipoxygenase pathways. *Curr. Neuropharmacol.* **18**, 885-895 (2020).

23. Zlotos, D. P., Jockers, R., Cecon, E., Rivara, S. & Witt-enderby, P. A. MT 1 and MT 2 melatonin receptors : Ligands , models , oligomers , and therapeutic potential. *J. Med. Chem.* **57**, 3161-3185 (2014).

24. Pérez-Sen, R. Queipo, M. J., Gil-Redondo, J. C., Ortega, F., Gómez-Villafuertes, R., Miras-Portugal, M.T. & Delicado, E. G. Dual-specificity phosphatase regulation in neurons and glial cells. *Int. J. Mol. Sci.* **20**, 1–23 (2019).

Table S3. Results of the *C. elegans* assays related to protein toxicity with mean values and standard deviations.

	**Concentration**	**Paralysis Assay**	**α-Synuclein Assay**	**MT Assay**
		**paralyzed on day 2 (%)**	**Fluorescence (% of vehicle control)**	**Fluorescence (% of positive control)**
Vehicle ctrl	1% DMSO	83.9 ± 2.3	100 ± 20	-8.6 ± 6.9
Levodopa (pos ctrl PD)	2 mM	N.T.	-14.6 ± 23.8**	N.T.
Quercetin (pos ctrl AD)	10 μM	69.5 ± 9.3	N.T.	N.T.
$ZnSO_4$ (pos ctrl MT)	10μM	N.T.	N.T.	100 ± 29.4***
Nemorosine A (**1**)	10 μM	73.8 ± 5,7	-23.0 ± 39.5**	81.1 ± 9.8**
	100 μM	51.0 ± 11.2*	36.2 ± 25.9	14.0 ± 30.28
Fargesine (**2**)	10 μM	61.0 ± 13.8	-28.7 ± 10.7**	182.0 ± 30.6***
	100 μM	70.8 ± 7,3	48.1 ± 48.0	87.1 ± 21.1**

N.T.: not tested. * $p<0.05$; ** $p<0.01$; *** $p<0.001$
